# Supplementary material for: Fatigue and associated factors among adult cancer patients receiving cancer treatment at oncology unit in Amhara region, Ethiopia
Source: PLoS One. 2023 Jan 6;18(1):e0279628. doi: 10.1371/journal.pone.0279628 (PMC9821493; doi:10.1371/journal.pone.0279628)
Supplement: Attachment — Submitted filename: Authors point by point Response.docx [file pone.0279628.s002.docx]

**Author’s Point-by-Point Response to the Reviewer's and Editors Reports**

**Fatigue And Associated Factors Among Adult Cancer Patients Receiving Cancer Treatment At Oncology Unit In Amhara Region, Ethiopia**

Corresponding Authors Dejen Tsegaye/ [dejenetsegaye@gmail.com](mailto:dejenetsegaye@gmail.com)

**Point by point response to Reviewers and Editors**

First and foremost, the authors would like to express their gratitude to the PLOSE ONE Journal editors and reviewers for thoroughly evaluating this work and offering the required corrections. We made changes based on the feedback we received and presented each comment point by point. The authors attempted to address all of the concerns expressed by the editorial board and reviewers. Please note that the response was written in blue font.

**Authors' responses to the authors' remarks**

**REVIEWER #1**

**Comment: Title:**  Appropriate and descriptive but I would prefer that the year should be removed from the title.

**Response:** We appreciate your input, and we removed the year as a result.

**Abstract:**

**Comment:** “Fatigue among cancer”: I think it should be fatigue among cancer patients. kindly revise

**Response:** We made the necessary corrections on the sentence in general. I'm grateful.

**Comment: Results:** with non-response rate of 9: what is the number 9? The rate is usually not a crude number it should be related to denominator as 9% or 9/1000 patients

**Response:** The non-response rate, as shown in brackets, is 1.97%. The number "9" represents the number of respondents who did not respond. I appreciate your thoughtful perspective. We made the required correction.

**Comment:** Line 3 in the results: “physical inactive “ should be replaced with physical inactivity

**Response:** Thank you! We made the necessary change.

**Comment:** Kindly remove redundancy. The conclusion includes repetition of the results.

**Response:** Thank you for your suggestion. We removed the repeated part.

**Introduction:** Well written and descriptive.

**Response: Thank you!**

**Material and methods:**

**Comment:** Line 67: I think study area should be replaced with setting

**Response:** Thank you, we replaced area with setting. Setting is the appropriate word.

**Comment: Population:** the authors should simply mention that the eligible criteria is cancer patients receiving treatment. kindly remove redundancy.

**Response:** Thank you for your comment. We made the necessary change.

**Comment:** Line 84: “and” should be added before hearing or intellectually impaired

**Response:** Thank you for your suggestion. We made the necessary change.

**Comment:** Table 1 is not cited in text.

**Response:** Thank you for your suggestion. We made the necessary change.

**Comment:** It is not clear why the sample size was multiplied by 1.5.

**Response:** As stated in a sentence in line "129," design effect has to be used in this study due to the variety of study settings and population types. Therefore, we multiplied the calculated sample size by 1.5 in order to have a sufficient sample size. I appreciate your asking for further information on this.

**Comment:** Sapling procedure is not clear. why stratified sampling was used ? what is the factor used for stratification?

**Response:** I appreciate you asking. Stratification was discovered to be necessary in this investigation due to variations in participants' provinces of residence.

**Comment:** Operational definition: it would be better if summarized in a table

**Response:** Thank you for your suggestion. We summarized the operational definition in a table.

**Comment:** How were all these questionnaires filled? Was that by face-to-face interview? How long did it take from each patient to film all these questions?

Response: Face-to-face interviews, which last 30 minutes for each patient, were used to complete the questionnaire. We explained in the ‘’ Data collection tools and personnel’’ part. Thank you for helping us to add clarification on this part.

**Results:**
**Comment:** Line 313: on why p-values was <0.25. selected to select variable?

**Response:** We chose 0.25 since there were a lot of variables included in the multivariable analysis with a p value of 0.25. We would have used 0.3 if the number of variables had been lower.

**Comment:** Line 334: it should be table 4 and should be cited in text.

**Response:** It is table 5, and we mentioned it correctly. Due to its automatic citation, the error was made when it was transformed to pdf. I appreciate your thoughtful feedback.

**Comment:** All data mentioned from line 319 to 333 are repeated in the table. Please remove unnecessary repetitions.

**Response**: I appreciate the comment. We decided to leave it as-is because the text portion interprets the table. We don't think all readers will be able to comprehend or interpret the table in the same way.  We will talk more and take the text portion out. I hope you will include your final observation or recommendation in the reviewer's second comment. Again, I'm grateful.

**Comment:** Figures are not cited in text.

**Response:** There are four figures incorporated in this study and Figure 1, 2, 3 and 4 are cited in line 131, 156, 273 and 283 respectively. Of these, figure 2 was missed. Thank you for the thoughtful comment.

**Comment:** Figures numbering is not correct

**Response:** After we correct the citation of figures accordingly, numbering is also corrected. Thank you for the comment.

**Discussion:**
**Comment:** Well written.

**Response:** Thank you!

**Comment:** The authors did not mention the limitations of their study.

**Response:** We mentioned our limitation in the study. Thank you!

**We appreciate all of the reviewers' and editors' helpful feedback, suggestions, and questions.**

**Thank you,**

**With kind regards!**
